# Imaging photoplethysmography as an easy-to-use tool for monitoring changes in tissue blood perfusion during abdominal surgery

**DOI:** 10.1038/s41598-022-05080-7

**Published:** 2022-01-21

**Authors:** Alexei A. Kamshilin, Valery V. Zaytsev, Alexander V. Lodygin, Victor A. Kashchenko

**Affiliations:** 1 Laboratory of New Functional Materials for Photonics, Institute of Automation and Control Processes, FEB RAS, Vladivostok, Russia; 2 First Surgical Department, Sokolov’s North-Western District Scientific and Clinical Center of the Federal Medical and Biological Agency, Saint Petersburg, Russia; 3 Department of Faculty Surgery, St. Petersburg State University, Saint Petersburg, Russia

**Keywords:** Cancer, Prognostic markers, Gastrointestinal cancer, Imaging and sensing

## Abstract

Evaluation of tissue perfusion at various stages of surgery is of great importance for the implementation of the concept of safe surgery, including operations on the abdominal organs. Currently, there is no accurate and reliable intraoperative method for assessing tissue perfusion that could help surgeons determine the risks of ischemia and improve outcomes. We propose novel method of intraoperative assessment of tissue perfusion using video camera synchronized with the electrocardiogram. The technique is referred to as imaging photoplethysmography (iPPG). It can be used continuously for monitoring blood supply to organs e.g., before and after anastomosis. In our study, we followed 14 different surgical cases (four stomach and ten colorectal cancers) requiring reconstruction of various organs with anastomosis. With iPPG, intraoperative blood perfusion was successfully visualized and quantified in all 14 patients under study. As most indicative, here we describe in detail two clinical demonstrations during gastrectomy for gastric cancer and right-sided hemicolectomy for cancer of the ascending colon. Feasibility of the iPPG system to assess blood perfusion in organs before and after anastomosis during open surgery was demonstrated for the first time.

## Introduction

Assessment of the parameters of blood circulation and tissue perfusion is extremely important during surgical interventions, including operations on the abdominal organs. The blood supply to organs can change in various pathological processes and/or be impaired due to surgical manipulations. Under these conditions, quantification of tissue perfusion can provide a surgeon with additional information to assist in planning and adjusting the surgical procedure. In particular, the ischemia factor is one of the most significant in the structure of the probable causes of the development of anastomoses leakage in abdominal surgery. The practical value of assessing this factor is determined by the possibility of intraoperative impact: the surgeon can change the level of resection in case of quantitative assessment of tissue perfusion. Various clinical signs have been suggested for intraoperative assessment of tissue perfusion, including bowel serosal color, vascular pulsation, and tissue bleeding. However, these signs are subjective and may lead to misinterpretations especially in visceral obesity or damage to the serous cover of an organ^1^.

In recent decades, there have been rather successful attempts to use the fluorescence technique in association with indocyanine green (ICG) fluorophore for intraoperative assessment of tissue perfusion both before and after anastomosis^2–4^. Application of this technology in clinical practice could help surgeons to optimize the level of resection and validate anastomosis. However, this method has disadvantages. To assess fluorescence, it is necessary to inject intravenous ICG solution. Repeated injections of ICG are characterized by a smaller increase in fluorescence intensity against the background of residual fluorescence after previous injections.

In addition, there are two optical methods exploiting coherent (laser) light that are considered promising for intraoperative assessment of blood flow parameters: laser Doppler perfusion imaging (LDPI) and laser speckle contrast imaging (LSCI). LDPI assesses spatial distribution of red-blood-cells velocity but it is a rather expensive system with low temporal resolution^5^. Moreover, the blood flow in laser Doppler methods is characterized by means of relative perfusion units, the physical significance of which has a contentious interpretation^6^. Another optical technique, LSCI, also referred to as dynamic light scattering, provides imaging of cerebral blood flow in two dimensions with high resolution^7,8^. More advanced modifications of LSCI technique, dynamic and multi-exposure, allow visualization of blood flow in the full field of view but require the use of high-speed video cameras^9^ Although speckle contrast values indicate the level of motion in tissue, they are not directly proportional to red blood cell velocity or blood flow^10^. Moreover, the results of the LSCI technique are ambiguous in interpretation due to multiple light scattering in biological tissue^9^.

Recently, an imaging photoplethysmography (iPPG) technique has been proposed for visualization and contactless quantification of tissue blood perfusion^11,12^. Unlike traditional ICG-based intraoperative imaging, iPPG does not require any intravenous administration of any substance. Successful application of iPPG at green illumination to assess changes in cortical blood-flow during open brain neurosurgery has been recently demonstrated in our group^11^. However, the use of iPPG in abdominal space is a challenging task due to significant variations of the examined organs morphology caused by respiration and peristalsis. These variations lead to motion artifacts that are many times greater than the sought changes in reflected light intensity caused by blood pulsations. As known, the main concern with iPPG is robustness to subject motion^13,14^, which becomes even more serious in the case of variable geometry of the tissue under study. Various algorithms for processing video data have been proposed, aimed at increasing the reliability of revealing the heartbeat related information in conditions of significant interference from motion artifacts. These algorithms include image stabilization techniques via two-dimensional correlation tracking^13^ or feature-related optical tracking^15^, use of Independent Component Analysis to retrieve a clean signal from color-channels mixture^14,16,17^, and stabilization of segmented images using optical flow algorithm to compensate heterogeneous and multidirectional motion^11,18^. However, most of the studies carried out with iPPG have dealt with images of subjects' skin. To the best of our knowledge, there is no reports related to assessment of blood perfusion in the abdominal organs by means of iPPG.

The aim of our study was to demonstrate feasibility of application of multimodal approach (synchronous recording of video frames of the tissue under study and patient’s electrocardiogram, ECG) to assess blood perfusion in organs before and after anastomosis during open surgery. The presented technique is a proof-of-concept that has been validated in real clinical conditions.

## Results

To estimate the potential of the iPPG system for intraoperative visualization of blood perfusion to various digestive organs under severe conditions of gastrointestinal surgery, we followed 14 different surgical cases requiring reconstruction of various organs with anastomosis. The basic operative maneuver in these cases was mesenteric dissection, which required devascularization, i.e., ligation of supplying blood vessels to the level of the intended anastomosis. It leads to blood supply redistribution throughout the organ, thus often revealing tissue perfusion disorders. First, a surgeon, from his own experience, determined different zones of the blood perfusion level in the organ to be prepared for surgery. He identified and marked several (up to three) areas of good, poor and absent blood supply. Then, the blood perfusion was measured by the iPPG system and compared with the surgeon’s evaluation. Note that the measurement results did not influence the surgeon’s decision on the level of resection. After anastomosis, blood perfusion in the connected organs was measured by iPPG to assess spatial distribution of the blood supply to the tissues of the joined organs.

In our study, various types of surgeries and anastomoses were monitored. Among them formation of a Roux anastomosis during gastroenterostomy, formation of an anastomosis with right-sided hemicolectomy, the descending colon with left-sided hemicolectomy, sigmoid colon resection, anterior (including low) rectal resection, and continuity restoration after Hartman procedure with formation of a descendo-rectal anastomosis. In all cases, the distribution of blood-flow parameters in the organs under study was successfully visualized by the iPPG system. This is spatial distribution of an amplitude of the pulsatile component (APC) of the photoplethysmographic (PPG) waveform, which allows us to visualize the blood supply to organs. The details of calculating the APC parameter are described below in the “Methods and patients” section. It was shown that this parameter is a measure of tissue perfusion^19–21^, and can be referred to as the perfusion index. Medical diagnoses, types of surgery, and estimated perfusion indices are listed in Table 1 for all 14 patients.

**Table 1:** Surgical cases monitored by the iPPG system.

#	Surgery	Diagnosis	*Perfusion index (%)		Measurement distance (cm)
			Panel B	Panel E	
Fig. 1	Total gastrectomy	Gastric cancer	2.64 ± 1.09	1.89 ± 0.50	35
Fig. 2	Laparoscopically assisted right-hemicolectomy	Ascending colon cancer	1.17 ± 0.70	1.26 ± 0.64	25
Fig. S1	Laparoscopic distal subtotal gastric resection	Gastric cancer	1.42 ± 0.87	1.18 ± 0.52	40
Fig. S2	Total gastrectomy	Gastric cancer	1.16 ± 1.22	2.06 ± 1.34	25
Fig. S3	Laparoscopically assisted right-hemicolectomy	Ascending colon cancer	0.88 ± 0.36	1.53 ± 0.87	30
Fig. S4	Laparoscopically assisted sigmoid colon resection	Sigmoid colon cancer	0.44 ± 0.37	0.53 ± 0.49	35
Fig. S5	Right hemicolectomy	Ascending colon cancer	1.27 ± 0.64	1.80 ± 1.17	25
Fig. S6	Laparoscopically assisted left-hemicolectomy	Descending colon cancer	1.03 ± 0.51	0.61 ± 0.27	25
Fig. S7	Total gastrectomy	Gastric cancer	1.09 ± 0.60	1.39 ± 0.60	35
Fig. S8	Laparoscopically assisted right-hemicolectomy	Cancer of ascending colon	1.07 ± 0.62	0.89 ± 0.67	30
Fig. S9	Laparoscopically assisted left colon resection	Sigmoid colon cancer	0.64 ± 0.40	0.62 ± 0.35	35
Fig. S10	Laparoscopically assisted sigmoid colon resection	Sigmoid colon cancer	0.43 ± 0.19	0.45 ± 0.20	25
Fig. S11	Laparoscopically assisted anterior rectal resection	Upper rectal cancer	0.43 ± 0.30	0.31 ± 0.20	40
Fig. S12	Descendorectostomy with ileostomy and hepatic resection	Rectosigmoid cancer	1.27 ± 1.77	1.55 ± 2.58	35

*Perfusion index is expressed as mean ± standard deviation.

**Figure 1:**
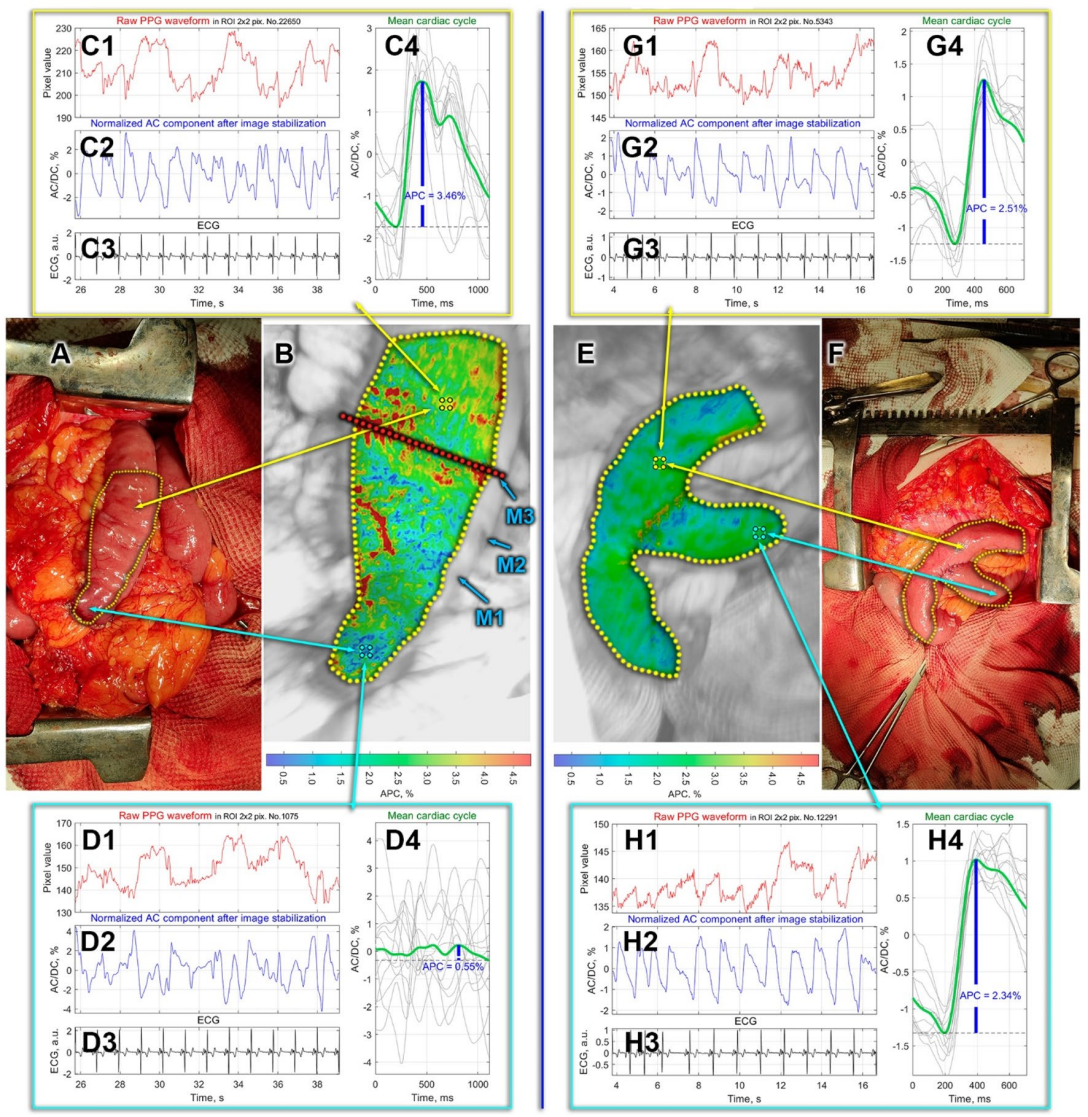
Intestinal blood perfusion in the case of T-shaped anastomosis. Panels (**A**,**F**) are photographs of the patient’s bowels before and after jejunojejunostomy, respectively. Yellow dashed lines in these panels mark the areas within which mapping of the perfusion was assessed. Spatial distributions of the amplitude of the pulsatile component (APC) before and after anastomosis are shown in Panels (**B,E**), respectively. APC maps are overlaid on the respective intestine images. The boundaries of zones with different levels of perfusion according to the surgeon's assessment are designated as M1–M3. The red dashed line in panel (**B**) indicates the final dissection line. The color bars in the bottom of Panels (**B,E**) show the APC as a percentage: more reddish, more perfusion. Panels (**C,D**) and (**G,H**) demonstrate PPG pulses in selected Region-Of-Interest (ROI sizing 2 × 2 pixels or 0.04 × 0.04 mm^2^) before and after anastomosis, respectively. Each ROI in which we show the PPG waveforms is centered on either yellow or cyan circle marked on APC maps. There are four graphs in each panel designated as X1, X2, X3, and X4 (X = A, C, G, or H). Graph X1 shows PPG waveform calculated in the selected ROI without image stabilization. X2 is the ratio of alternating and slow varying components of the PPG waveform in the same ROI after image stabilization, law-pass filtering, and signal inversion. X3 is ECG signal synchronously recorded with video frames. Graph X4 shows one-cardiac-cycle pulse wave (thick green line) obtained after averaging of particular pulse waves (thin gray lines) over 12 cardiac cycles.

**Figure 2:**
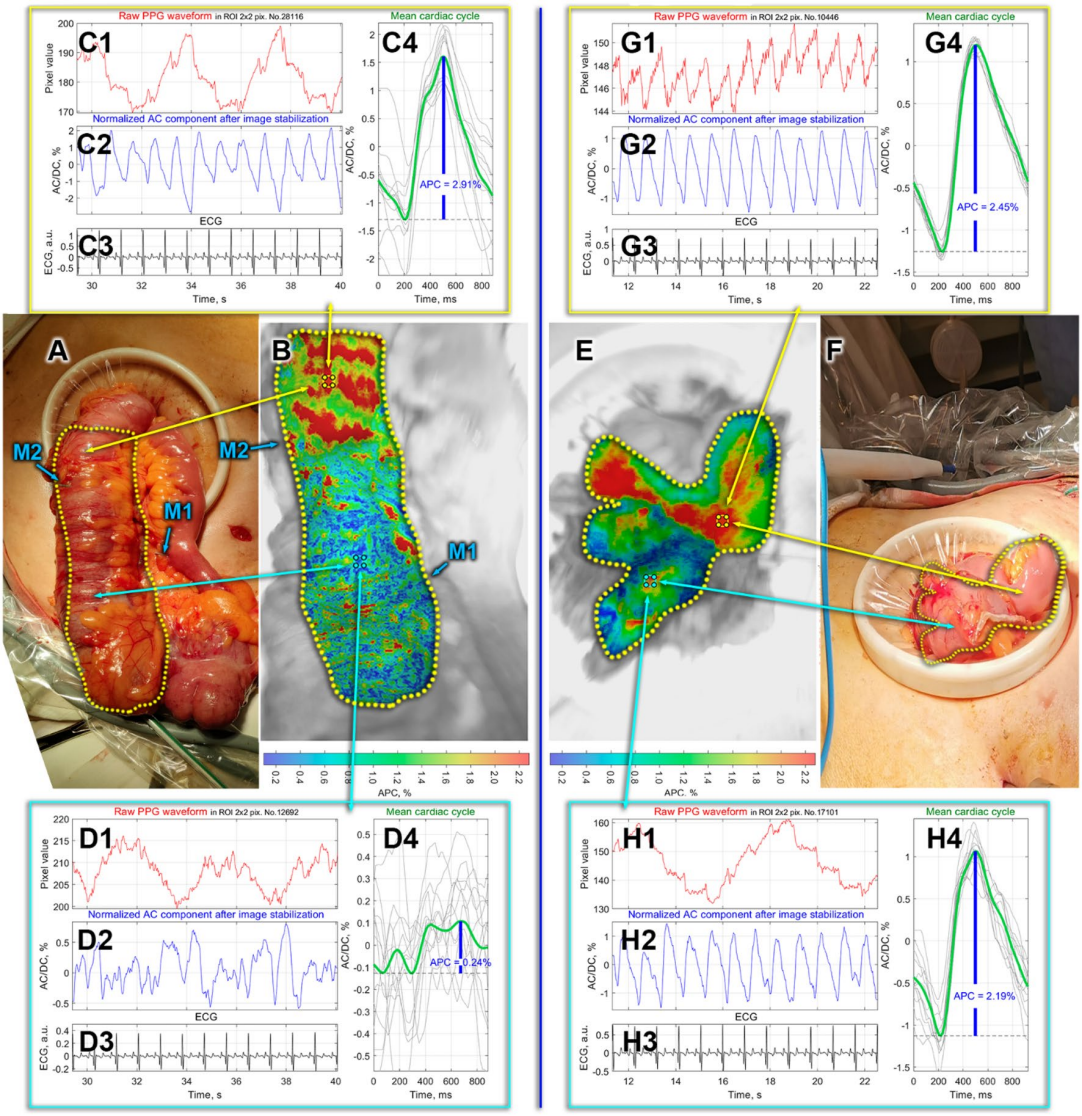
Intestinal blood perfusion in the case of side-to-side anastomosis. Panels (**A,F**) are color photographs of patient’s intestines before and after anastomosis, respectively. Spatial distributions of the APC before and after anastomosis overlaid on the respective intestine images are shown in Panels (**B,E**), respectively. Points M1 and M2 mark the boundaries of visible vascularization and mesenteric attachment, respectively. The color bars in the bottom of Panels (**B,E**) show APC percentagewise. Panels (**C,D**) and (**G,H**) demonstrate PPG pulses in selected ROIs before and after anastomosis, respectively. The meaning of the graphs in these panels is the same as in Fig. 1.

Each row in the Table 1 refers to a certain surgical case and is numbered according to the number of figure in which the assessed perfusion maps are shown. While Figs. 1 and 2 are presented in the main text, Fig. S1 through Fig. S12 are in the Supplementary file. Table 1 lists the diagnosis and type of surgery for each patient. The perfusion index is shown as the APC averaged over the area within which mapping of blood flow parameters was assessed. In Table 1, we show two perfusion values for each case: assessed before anastomosis or physiological test (marked as Panel B in the figures) and thereafter (marked as Panel E). The last column shows the distance between the iPPG module and a tissue under study. As one can see, the mean perfusion index of organs varies greatly from one patient to another: from 0.31 to 2.64%. At the same time, we observed good reproducibility of quantitative APC assessment for the same organ with extended photoplethysmography recording. It is worth noting that after the anastomosis was applied, the mean perfusion index increased in five cases (Fig. 2, Fig. S2, Fig. S3, Fig. S5, and Fig. S7), and decreased in four cases (Fig. 1, Fig. S1, Fig. S6, and Fig. S8). In other cases, no statistically significant changes were found. As a demonstration of the potential of iPPG in assessing tissue perfusion, we present in detail two of the most representative cases below.

### The case of T-shaped Roux-en-Y anastomosis

The first case is presented by measurements of blood-circulation parameters during gastrectomy for gastric cancer. After stomach removal and lymph node dissection (removal of regional lymph nodes), restoration of the gastrointestinal tract was performed. The reconstruction involved dividing the proximal part of the jejunum into two loops that formed the letter Y (Roux-en-Y reconstruction). One part (proximal) of the jejunum draining the biliary tract and pancreas was designated as a biliopancreatic loop while the other part (anastomosed with the esophagus) as the alimentary loop. Before the jejunum was cut 20 cm from Tretz's ligament, the mesentery was transected in this zone with ligation and crossing of the second jejunal branch in order to sufficiently mobilize the future alimentary loop. Moreover, to create an optimal space for using the stapler, additional devascularization of a jejunum segment was performed. As a result of these surgical procedures, a segment of the jejunum was formed with a difference in perfusion from complete cessation of blood supply to normal blood supply. At this stage, the surgeon identified several zones with different perfusion by placing three marks. A color photograph of the jejunum prepared for anastomosis is shown in Fig. 1A. The surgeon’s markings are shown in the enlarged view of the jejunum (Fig. 1B) as M1, M2, and M3. According to the surgeon, perfusion below M3 is good, M2 marks the center of the region with intermediate perfusion, whereas the area above M1 is not perfused.

The surgeon’s prognosis was compared with spatial distribution of the APC parameter assessed by the iPPG system. APC map in Fig. 1B is overlaid with the enlarged image of the jejunum. It is coded in pseudo colors with the scale shown on the right: red for more perfusion and blue for less. As seen in Fig. 1B, perfusion is higher in the lower jejunum, consistent with the surgeon's prognosis. To quantify the change in perfusion index along the jejunum, we estimated the mean APC in several large ROIs of 60 × 60 pixels. The assessment showed that the maximum perfusion index (100%) is observed around ​​the yellow circle, then decreasing to 58% near the M1 mark, to 37% near M2, and to 12% near M3. Moreover, PPG pulses (see details of their calculations in the subsection “Data Processing”) in the lower part displays poor synchronization with ECG (shown in the panel D, graph D4 in Fig. 1) that is usually observed with impaired circulation. In contrast, there is reasonable synchronization of ECG and PPG waveform in the upper part of the jejunum as illustrated in the graph C4, panel C in Fig. 1). The parameters of blood supply to the jejunum, measured with iPPG, confirmed the judgment of the surgeon, who decided to perform a resection near the M3 marker along the red dashed line.

The formation of the interintestinal Roux-en-Y anastomosis was carried out according to the "end-to-side" type: by suturing the end of the biliopancreatic loop into the side of the continuation of the alimentary loop. It was done manually using a two-row continuous stitch with PDS 3-0 suture. After the anastomosis has been performed, the tissue blood supply to the connected organs was assessed again by the iPPG system. Color photograph of a general view of the connected intestines is shown in Fig. 1F. The spatial distribution of the APC perfusion index overlaid with an enlarged image of the anastomosis is shown in Fig. 1E. As seen, the perfusion index is much more evenly distributed over both connected organs. Additionally, the mean PPG pulses are well synchronized over almost the entire surface of both intestines, as shown by the examples of calculating these pulses 40 × 40 μm^2^ regions located in the centers of the red and cyan squares (see graphs G4 and H4 in Fig. 1G,H, respectively). Mean PPG pulse shown in Fig. 1G can be considered as a reference, since there were no factors compromising blood supply in this part of the jejunum. The area for measuring the perfusion in the biliopancreatic loop after the anastomosis formation (Fig. 1H) corresponds to the area in which perfusion was estimated before the anastomosis (Fig. 1C). In this area after anastomosis, good perfusion (APC = 2.49 ± 0.42%) is observed despite a decrease in the perfusion index (APC = 2.77 ± 0.76% before anastomosis). The good blood supply to the anastomosed organs, observed in this case, gives hope for the success of the particular surgical intervention.

### The case of ileotransverse anastomosis

This example presents the assessment of tissue perfusion during the reconstructive stage after right-sided hemicolectomy for cancer of the ascending colon. A laparoscopic medial–lateral approach was used with the dissection, clipping and transection of the ileocolic, right colic vessels and right branches of the middle colic vessels with D3 lymphadenectomy. After mobilization of the right sections of the colon and terminal ileum, these sections together with the tumor were extracted through a minilaparotomic incision (5 cm) in the umbilical zone. Perfusion zones were formed because of ligation of regional vessels. Clips were placed on the ileum and transverse colon (M1 and M2 in Fig. 2A) marking the place of attachment of the mesentery with the main vessels and the border of visible vascularization, respectively. The area with significant decrease in perfusion was located at a distance of 3 cm from the visible edge of the mesentery.

After the aforementioned surgical preparation, the perfusion of the colon prepared for anastomosis was measured by the iPPG system. The distribution of the APC parameter is shown in Fig. 2B. It is clearly seen that in the upper part (according to the orientation of the photo in Fig. 2B) of the colon (above the M2 mark), the APC is significantly higher than in the lower part: 2.46 ± 0.38% versus 0.73 ± 0.45%. Moreover, one can see in Fig. 2D that in the lower part of the colon, the PPG pulses are completely out of sync with the ECG. In contrast, in the upper part of the colon, there is a confident synchronization of PPG and ECG, forming a typical shape of the mean PPG pulse (see Fig. 2C). This result is in good agreement with the surgeon's assessment of the blood supply.

After the iPPG measurements, both intestines were transected at the level (mark M2) near the border of the dissected mesentery with the vessels using a three-row linear stapler DST. Side-to-side antiperistaltic ileotransversal anastomosis was formed manually using a continuous two-row stitch with PDS 3–0 suture. The appearance of the formed anastomosis is shown in Fig. 2F. After the anastomosis formation, the iPPG measurement of tissue perfusion was performed again simultaneously on both components of the structure. APC mapping overlaid on the monochrome image of the anastomosis is shown in Fig. 2E. The ileal perfusion index was higher (2.35 ± 0.11%) than that of the transverse colon (1.59 ± 0.28%), which is visually expressed in a redder shade of the APC distribution in the ileum in Fig. 2E. At the same time, after anastomosis, excellent synchronization of PPG pulses and ECG is observed in both connected intestines, which is confirmed by Figs. 2G,H. Note that the mean perfusion index in the well-perfused area of the transverse colon before anastomosis (Fig. 2C) was higher than in the same area (Fig. 2H) after anastomosis: 2.38 ± 0.43% vs. 1.58 ± 0.28%. Despite the significant difference in the colon perfusion index before and after the anastomosis, the excellent synchronization of PPG and ECG allows us to hope for a favorable outcome of the surgery in this case, as well.

## Discussion

In our study, successful visualization and quantification of organ perfusion using simple ECG-synchronized video (iPPG system) was achieved in all 14 patients. In the main text we presented in detail two cases of tissue-perfusion assessment for different variants of gastrointestinal tract reconstruction. From the point of view of the research protocol design, we selected the cases of anatomically superficial anastomoses. This allowed us to carry out measurements both at the stage of organ mobilization and after suturing of tissues, in contrast to the esophagojejuno and transversorecto anastomoses that are more "risky" but anatomically difficult to access. All other cases are presented in the Supplementary Figures. One of the tasks of our study was to demonstrate feasibility of measurements of perfusion parameters of tissues by using the iPPG technique under the influence of various unfavorable factors that can affect the blood circulation of the organ. We assumed that the formation of anastomosis is one of the factors that can affect the perfusion of the surrounding tissues since we cross the intestine and cut off the blood supply before the tissues are sutured. In addition, the suture itself leads to greater tissue ischemia. If the initial blood supply to the tissues is compromised, anastomosing can reduce perfusion below a critical level and provoke anastomotic leakage.

Another factor, obviously more significant, is vascular ligation and organ skeletization. The point is that the operative technique for selected operations for cancer involves a central (close to the main vessel) ligation of blood vessels, which leads to a significant decrease or complete cessation of the blood supply to the removed complex of organs. Moreover, before transecting the intestine, the surgeon separates the mesentery with the vessels in the area of ​​the planned transection. This leads to the cessation of any blood circulation from the outside, with the exception of interstitial blood supply from the border of the proposed resection, which is able to provide the vital activity of tissues only in a limited area of about 2 cm. Therefore, in the complex of organs to be removed, an area is formed which suffers from a deficit of blood supply because of both the intersection of the regional feeding vessels and skeletization of the intestine. Moreover, the further the blood supply assessment point is located from the point of completion of skeletization, the lower the perfusion parameters will be. It is these measurements that were carried out in our study before the formation of the anastomosis. The iPPG system revealed good perfusion indices, moving away from the end of skeletization towards the intestine at the mesentery (Figs. 1C and 2C). Further, when the measurement zone is moved closer to the level of resection, a slight decrease in perfusion occurs, since the blood supply switches from bilateral to unilateral (from the part of the intestine with the mesentery). Moving the measurement zone along the skeletized part of the intestine even further from the resection line, we observe a progressive decrease in the perfusion parameters (Figs. 1D and 2D). Unfortunately, we cannot yet say what the specific value of the perfusion index is, which could be taken as the threshold below which the likelihood of developing adverse effects will be very high. However, this work clearly demonstrates the capabilities of the iPPG technique in quantitative intraoperative evaluation of perfusion, which fully correlates with the clinical assessment of tissues by the surgeon. It is worth noting that in our study the surgeon knew the level of tissue perfusion, while the iPPG technique revealed parameters consistent with the surgeon's assessment. The measurement results did not affect the surgeon's decision on the level of resection.

From a technical point of view, the most challenging was the use of the iPPG system in intraoperative conditions, when the organ under study could move several centimeters during video recording for 10–12 s, while changing its morphology. Nevertheless, the chosen method of synchronous recording of video frames and ECG, together with the original data processing method (described in the following “Methods and patients” section), has demonstrated its feasibility to visualizing and quantifying the perfusion of various organs.

## Methods and patients

### Patients

This study was performed within the first surgical department of the North-Western District Scientific and Clinical Center named after L.G. Sokolov of the Federal Medical and Biological Agency (Saint-Petersburg, Russia) in accordance with ethical standards presented in the 2013 Declaration of Helsinki. The study plan was approved by the Ethics Committee of the North-Western District Scientific and Clinical Center, decision No 7 of May 12, 2021 (prior of the study). All patients and/or their legal representatives provided informed consent in written form for participation in the study. All surgical interventions were performed according to the standard techniques in 14 patients with various types of gastric and colorectal cancer. Each surgical procedure consisted of two main stages: resection and reconstruction. The stage of resection consisted of ligation of the regional vessels nearby their origin and mobilization of the organ from which the tumor originates with its mesentery. Because of this maneuver, a significant decrease in the blood supply to the mobilized part of the organ was expected. The surgeon then dissected a limited part of the intestine near the intersection by cutting off the mesentery from the intestine (organ devascularization). After this maneuver, further disruption of the blood supply in the removable part of the gastrointestinal tract was expected. At the end of the resection stage, the blood supply to the part of the intestine assigned for removal could be carried out only through the vessels of the intestinal wall. This led to a significant difference in blood supply at the borders of the resection line from complete absence to good circulation comparable to the initial level, which was visualized by the iPPG system.

### Measuring system

**Figure 3:**
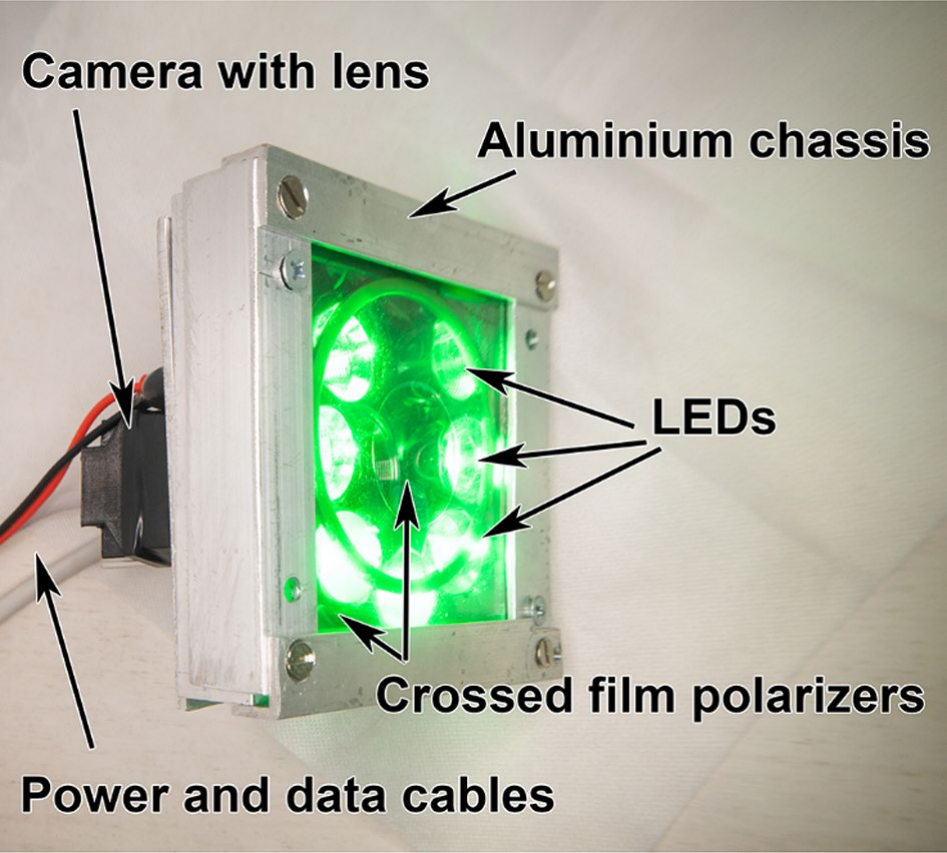
Custom-made iPPG system used in the experimental study.

Tissue blood perfusion was measured using a custom-made iPPG system synchronized with a digital electrocardiograph. The system design is similar to that described in our previous paper^18^. A photograph of the used iPPG module is shown in Fig. 3. The module includes a digital monochrome CMOS camera (8-bit model GigE uEye UI-5220SE-M-GL of the Imaging Development Systems GmbH) and an illuminator with 7 light-emitting diodes (LEDs, model BL-HP20APGCL-5W STAR). To increase the uniformity of illumination of the tissue under study, LEDs were assembled around the camera lens. They emitted green light at a wavelength of 535 nm. To maintain the operating temperature of the LEDs, the illuminator chassis was made of aluminum. The camera lens and LEDs were covered by polarizing films with mutually orthogonal orientation thus reducing influence of specular reflections and motion artefacts^22,23^. The iPPG module has been aligned so that the tissue surface normal is as close to the optical axis of the camera lens as possible. In this geometry, the effect of ballistocardiographic artifacts^16^ is significantly reduced. The distance between the camera lens and tissue under study was between 25 and 40 cm.

Video frames of the tissue were recorded at 39 frames per second with a resolution of 752 × 480 pixels and continuously stored in a personal computer. During the video recording, the operating room lighting was dimmed and a surgical light was turned off so that the illumination of the tissue by the iPPG illuminator exceeded the ambient illumination at least 50 times. To improve the accuracy of measuring blood pulsation and obtain reliable physiological results, we recorded video frames of the skin synchronously with the electrocardiogram (ECG). To record ECG and sync pulses from a video camera, we used a digital electrocardiograph (model KAP-01- “Kardiotekhnika-EKG,” Incart Ltd., St. Petersburg, Russia) with a sampling rate of 1 kHz, providing synchronization of frames and ECG with an accuracy of about 1 ms. At the stage of data processing, the ECG signal served as a cardiac timing reference.

### Study protocol

During perfusion measurements, the surgeon identified the organ or region of interest, and video recording of the selected area was carried out from 30 to 50 s. In some cases, the surgeon or his assistant held the surrounding tissues with their hands to improve the visible access to the organ under study. The IPPG module allowed an unlimited number of selected areas to be recorded.

### Data processing

Recorded video frames of tissues were processed together with ECG by using custom software implemented on the MATLAB® platform^18,21^. At the first step, digital stabilization of the tissue images was applied to minimize influence of motion artifacts. Considering that different parts of the tissue image are shifting stochastically and heterogeneously, each image frame was divided into 8 × 8 pixel segments, and the motion of each segment was compensated independently. It is assumed that variations in the light intensity measured by the camera's photosensitive matrix are determined by both changes in blood volume interacting with light and tissue movement. The motion-related component is proportional to the image gradient and lateral offset. This component was assessed in every segment by optical flow algorithm using gradient method^24^. After reconstruction, the motion-related component was subtracted from the original signal.

At the second step, the image frame was divided into small regions of interest (ROI) sizing 2 × 2 pixels, which is about 40 × 40 μm^2^ in the tissue plane. Each ROI was selected to share a common border with adjacent ROI without overlapping. For mapping blood perfusion parameters, a PPG waveform was calculated as frame-by-frame evolution of the average pixel value in every ROI. While the X1 graphs in Figs. 1 and 2 (as well as in the Supplementary Figures) show examples of the PPG waveforms assessed before the image stabilization step, to quantify the blood perfusion, we used waveforms calculated in the same small ROI after the motion artefacts removal. Examples of these waveforms in some selected ROIs are shown in the graphs X2. In contrast to X1 graphs, here we first averaging the ROI values within floating time window, equal to duration of the respective cardiac cycle assessed from the synchronously recorded ECG. This is so-called slowly varying DC component of the PPG waveform. Then, at each time point (for each frame), the current ROI value is divided (normalized) by the DC component thus emphasizing an alternating component (AC) of the waveform. Since both components are proportional to the incident light intensity^25,26^, the use of the AC/DC ratio compensates unevenness of the incident illumination. After subtracting the unity from the calculated AC/DC ratio and inverting its sign, we obtained a waveform that correlates positively with changes in arterial blood pressure in the nearest vessels^18,27^. These waveforms are shown in graphs X2 in all Figures including Supplementary. All PPG waveforms were filtered to remove high-frequency noise by means of a low-pass filter (4 Hz), which was implemented using the filtfilt function in Matlab® to perform zero-phase digital filtering of the waveforms.

Importance of the first step of data processing (image stabilization) is clearly recognized after comparing the PPG waveform in the same ROI before and after the motion artefacts removal (see, e.g., graph D1 versus graph D2 in Fig. 2). If in non-stabilized frames, modulation associated with the heart is practically not detected, then after stabilization it is clearly seen that PPG pulses follow R-peaks of ECG. Nonetheless, in the case of relatively high modulation of blood volume in the vessels (e.g., panel G in Fig. 2), a well-resolved heart related modulation is observed even in the unprocessed signal (graph G1 in Fig. 2).

At the third step, we assessed a mean PPG pulse for 12 subsequent cardiac cycles. To this end, the PPG waveform was cut into individual PPG pulses so that the beginning of each pulse coincided with the corresponding R-peak of the ECG, and then these pulses were ensemble averaged^18,21^. Any of X4 graphs in Fig. 1 or Fig. 2 exemplifies the mean-PPG-pulse assessment: individual PPG pulses are shown in light gray, and the mean pulse is shown in bold dark green. Comparing the mean PPG pulses in ROIs of different location, one can see that PPG pulses of individual cardiac cycles are not always well synchronized with each other. Examples of such desynchronization are graphs C4 in Fig. 1 and C4 in Fig. 2. Poor synchronization naturally decreases the amplitude of the mean pulse and accordingly decreases the amplitude of pulsatile component (APC), which was defined as the difference between the maximum and minimum values of the mean pulse PPG. Note that an index of PPG pulses synchronization can also serve as a measure of the local perfusion state^28^. Interestingly, in the case of a satisfactory blood supply, the criterion of PPG pulse synchronization turns out to be workable even with significant variability in the cardiac cycle duration. This is confirmed in Fig. 1G,H. In this patient at the time of recording, there was a significant variability in the cardiac cycle duration, which is visible with the naked eye in the graphs G3 and H3 in Fig. 1. Nevertheless, individual PPG pulses are reasonably well synchronized with each other, if their beginnings are aligned with the corresponding R-peaks. It should be noted that the position of the minima of the PPG pulses does not depend on the cardiac cycle duration, but is primarily determined by the time of arrival of the pressure wave to the point of measurement. Therefore, the variability of the cardiac cycle duration does not affect the synchronization of the individual PPG pulses. The time interval of 12 subsequent cardiac cycles defines the temporal resolution with which variations of APC can be assessed. It depends on the heart rate and may vary from 6 to 16 s. Processing time needed to assess the spatial distribution of the perfusion index (APC mapping) ranged from 40 to 70 s, depending on the size of the region selected for analysis.

## Supplementary Information

Supplementary Figures.
