# Supplementary material for: Imaging photoplethysmography as an easy-to-use tool for monitoring changes in tissue blood perfusion during abdominal surgery
Source: Sci Rep. 2022 Jan 21;12:1143. doi: 10.1038/s41598-022-05080-7 (PMC8782890; doi:10.1038/s41598-022-05080-7)
Supplement: Supplementary file 1 — Supplementary Figures. [file 41598_2022_5080_MOESM1_ESM.pdf]

Description. *In all Figures below, spatial distributions of the index perfusion (APC maps) are overlaid on the respective intestine images. The color bars in the bottom of the panels B and E show the APC as a percentage: more reddish, more perfusion. Panels C, D and G, H demonstrate PPG waveforms in selected ROIs sizing 2 × 2 pixels or 0.04 × 0.04 mm*^{2}*. Position of the selected ROI is indicated by either yellow or cyan circle: ROI is in the center of the circle. There are four graphs in each panel designated as X1, X2, X3, and X4 (X = C, D, G, or H). Graph X1 shows PPG waveform in a ROI calculated without image stabilization during 12 cardiac cycles. X2 is the ratio of alternating and slow varying components of the PPG waveform in the same ROI after image stabilization, law-pass filtering, and signal inversion. X3 is ECG signal synchronously recorded with video frames.*

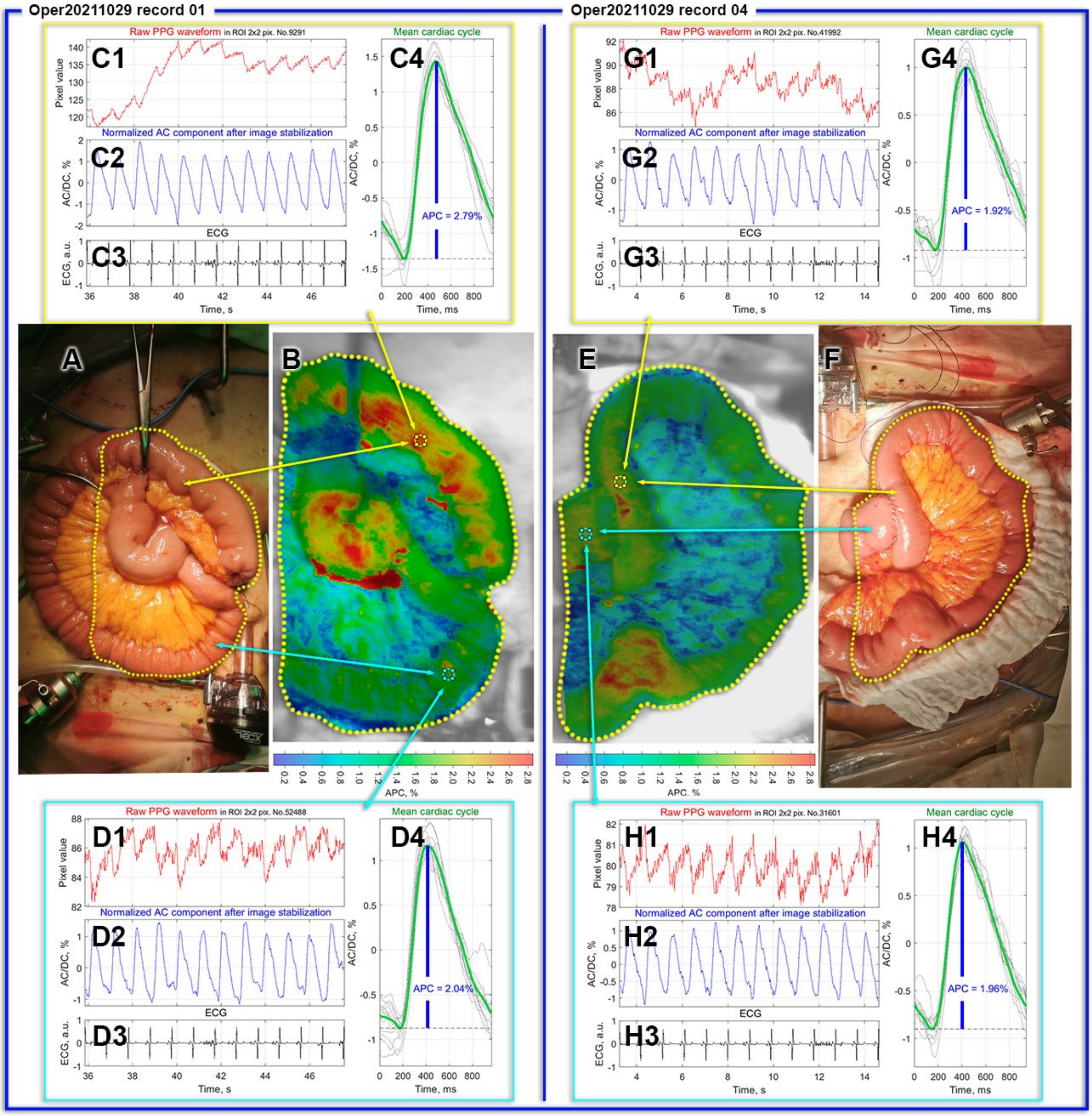

Figure S1. Blood perfusion visualization and examples of PPG waves. Diagnosis: Gastric cancer. Surgery: Laparoscopic distal subtotal gastric resection. Panels A and F are photographs of the patient's bowels before and after anastomosis, respectively. Yellow dashed lines in these panels indicate the areas within which mapping of the perfusion was assessed. Spatial distribution of the index perfusion APC before and after anastomosis are shown in Panels B and E, respectively.

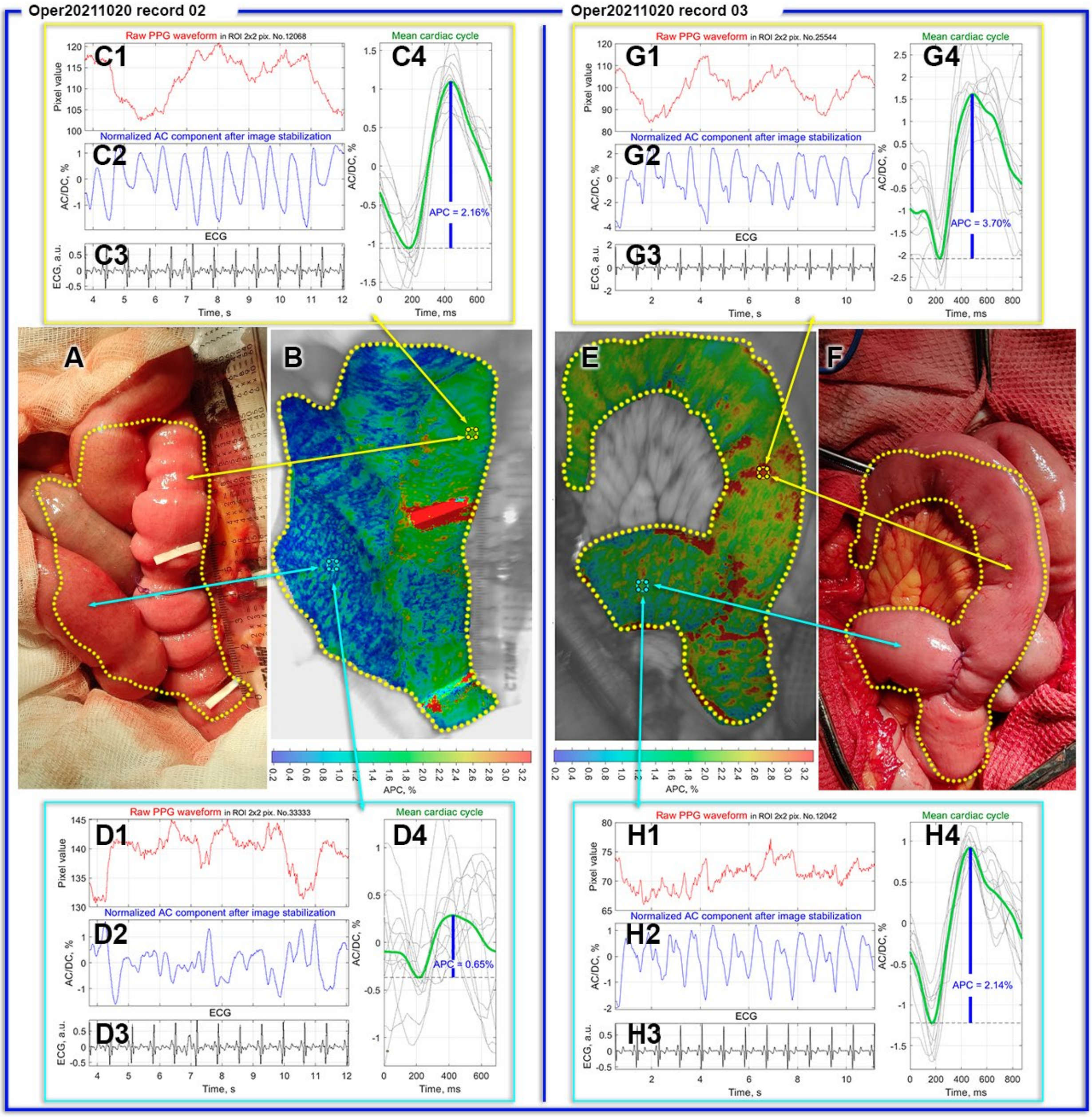

Figure S2. Blood perfusion visualization and examples of PPG waves. Diagnosis: Gastric cancer. Surgery: Total gastrectomy. Panels A and F are photographs of the patient's bowels before and after anastomosis, respectively. Yellow dashed lines in these panels indicate the areas within which mapping of the perfusion was assessed. Spatial distribution of the index perfusion APC before and after anastomosis are shown in Panels B and E, respectively.

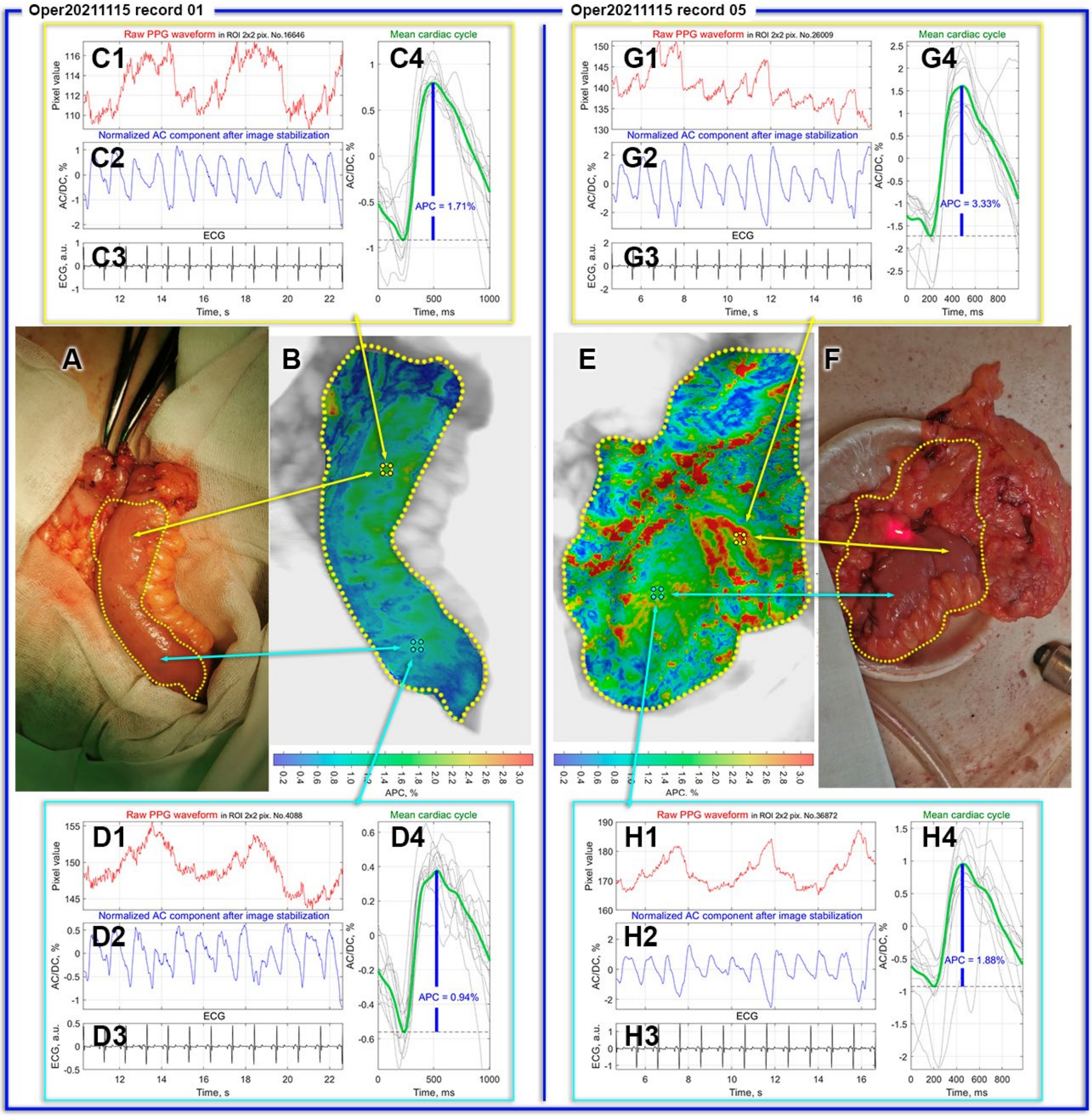

Figure S3. Blood perfusion visualization and examples of PPG waves. Diagnosis: Ascending colon cancer. Surgery: Laparoscopically assisted right-sided hemicolectomy. Panels A and F are photographs of the patient's bowels before and after anastomosis, respectively. Yellow dashed lines in these panels indicate the areas within which mapping of the perfusion was assessed. Spatial distribution of the index perfusion APC before and after anastomosis are shown in Panels B and E, respectively.

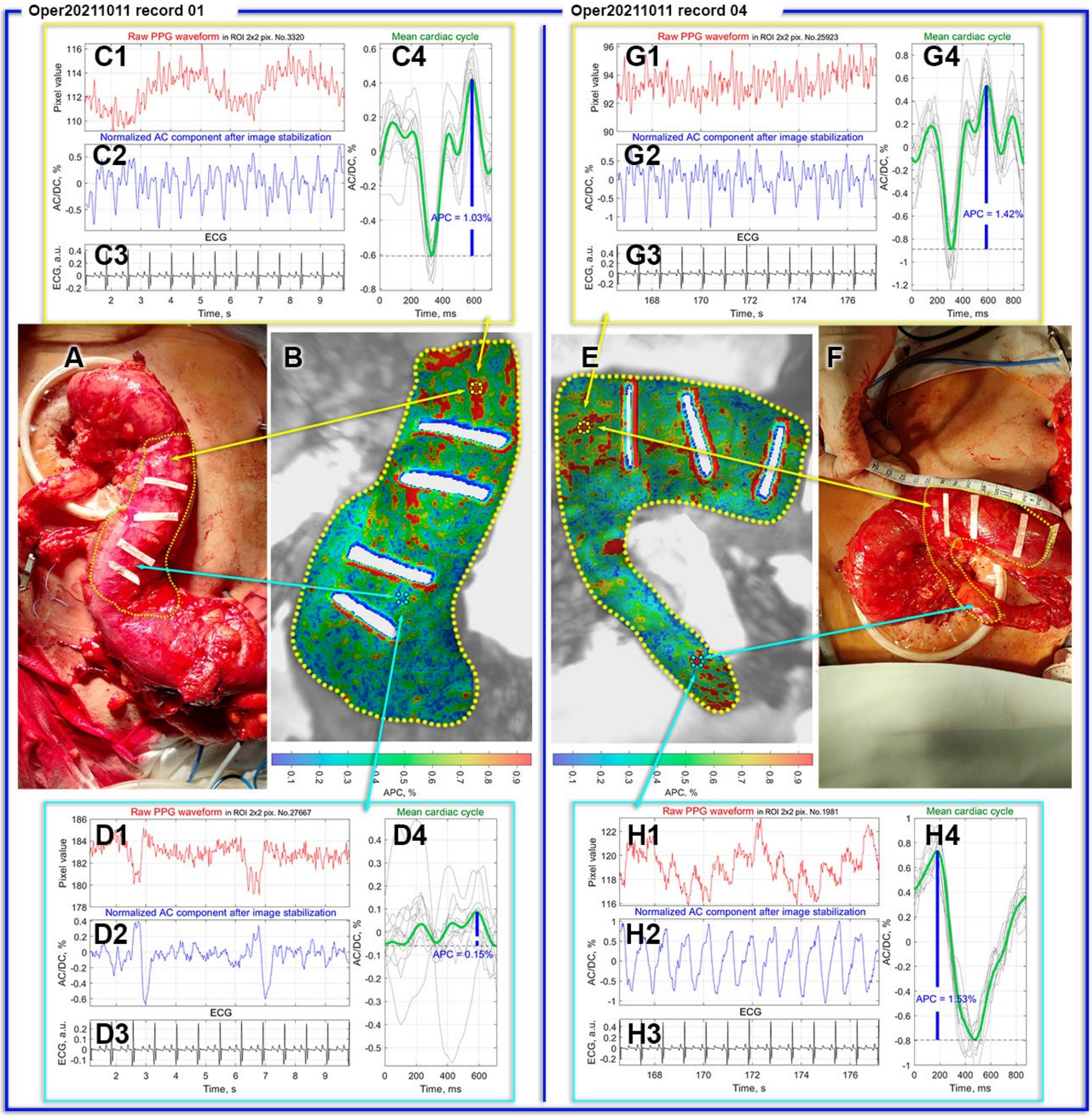

Figure S4. Blood perfusion visualization and examples of PPG waves. Diagnosis: Sigmoid colon cancer. Surgery: Laparoscopically assisted sigmoid colon resection. Panels A and F are photographs of the patient's bowels before and after anastomosis, respectively. Yellow dashed lines in these panels indicate the areas within which mapping of the perfusion was assessed. Spatial distribution of the index perfusion APC before and after anastomosis are shown in Panels B and E, respectively.

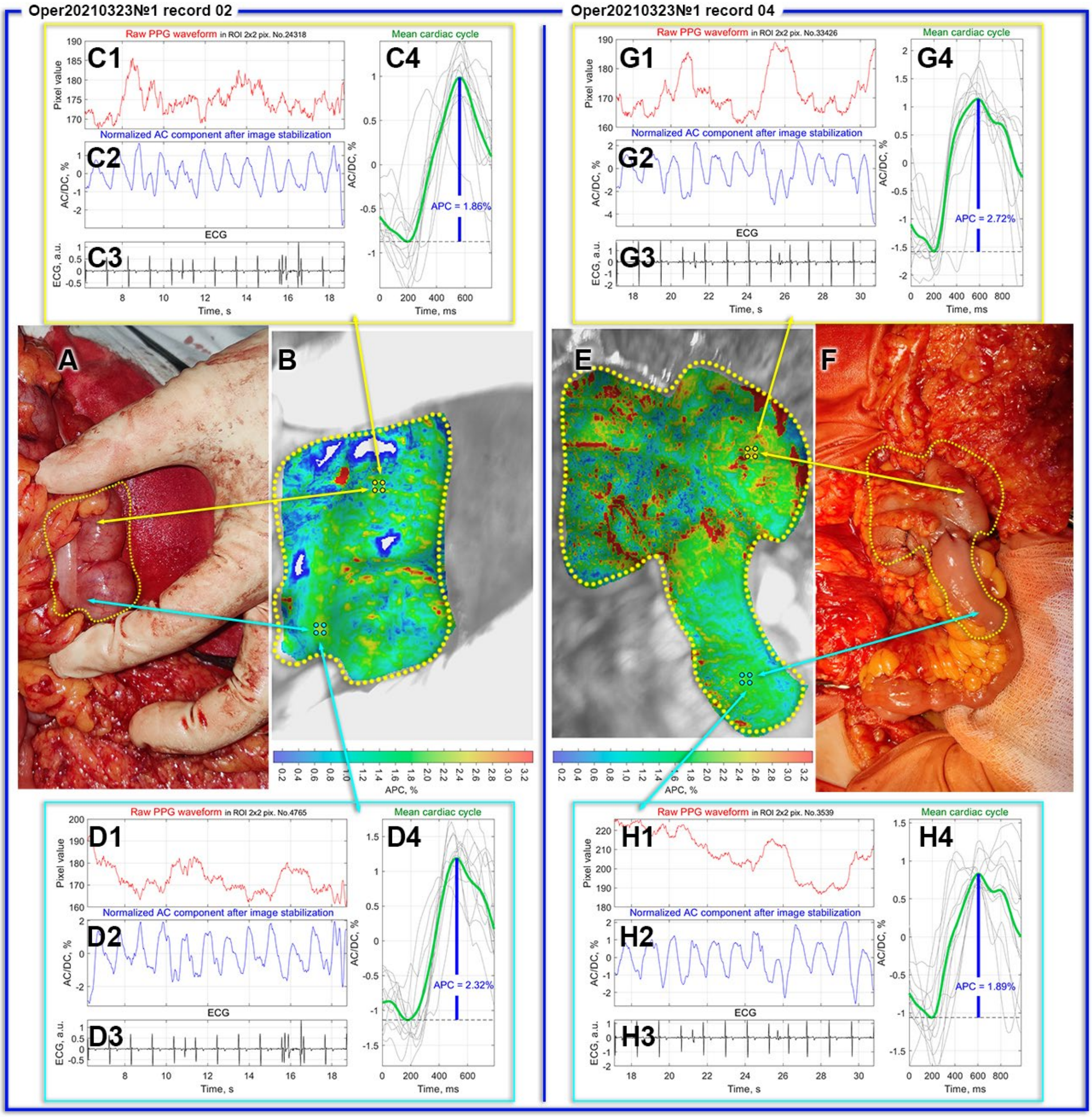

Figure S5. Blood perfusion visualization and examples of PPG waves. Diagnosis: Ascending colon cancer. Surgery: Right hemicolectomy. Panels A and F are photographs of the patient’s bowels before and after anastomosis, respectively. Yellow dashed lines in these panels indicate the areas within which mapping of the perfusion was assessed. Spatial distribution of the index perfusion APC before and after anastomosis are shown in Panels B and E, respectively.

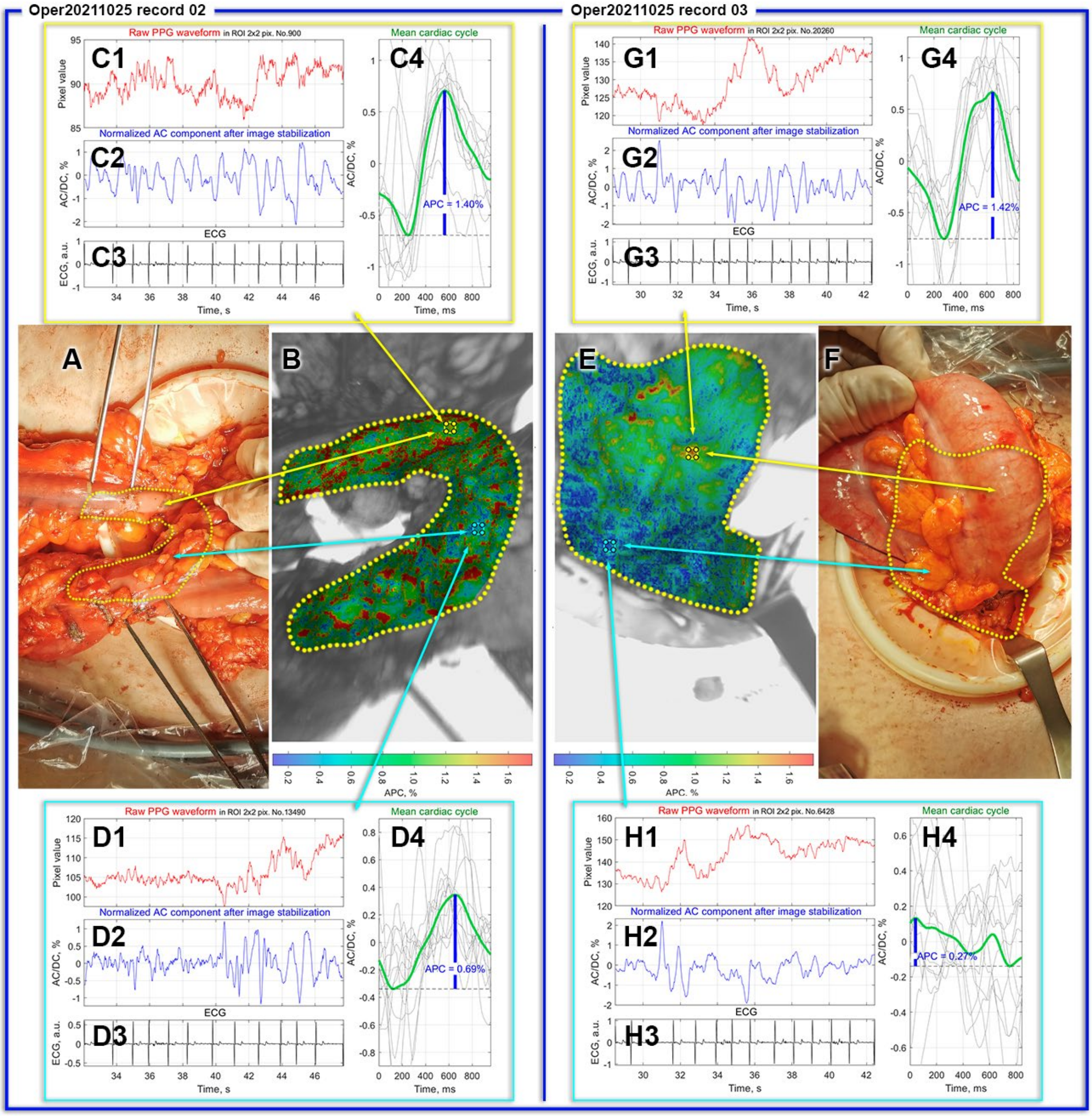

Figure S6. Blood perfusion visualization and examples of PPG waves. Diagnosis: Descending colon cancer. Surgery: Laparoscopically assisted left-sided hemicolectomy. Panels A and F are photographs of the patient's bowels before and after anastomosis, respectively. Yellow dashed lines in these panels indicate the areas within which mapping of the perfusion was assessed. Spatial distribution of the index perfusion APC before and after anastomosis are shown in Panels B and E, respectively.

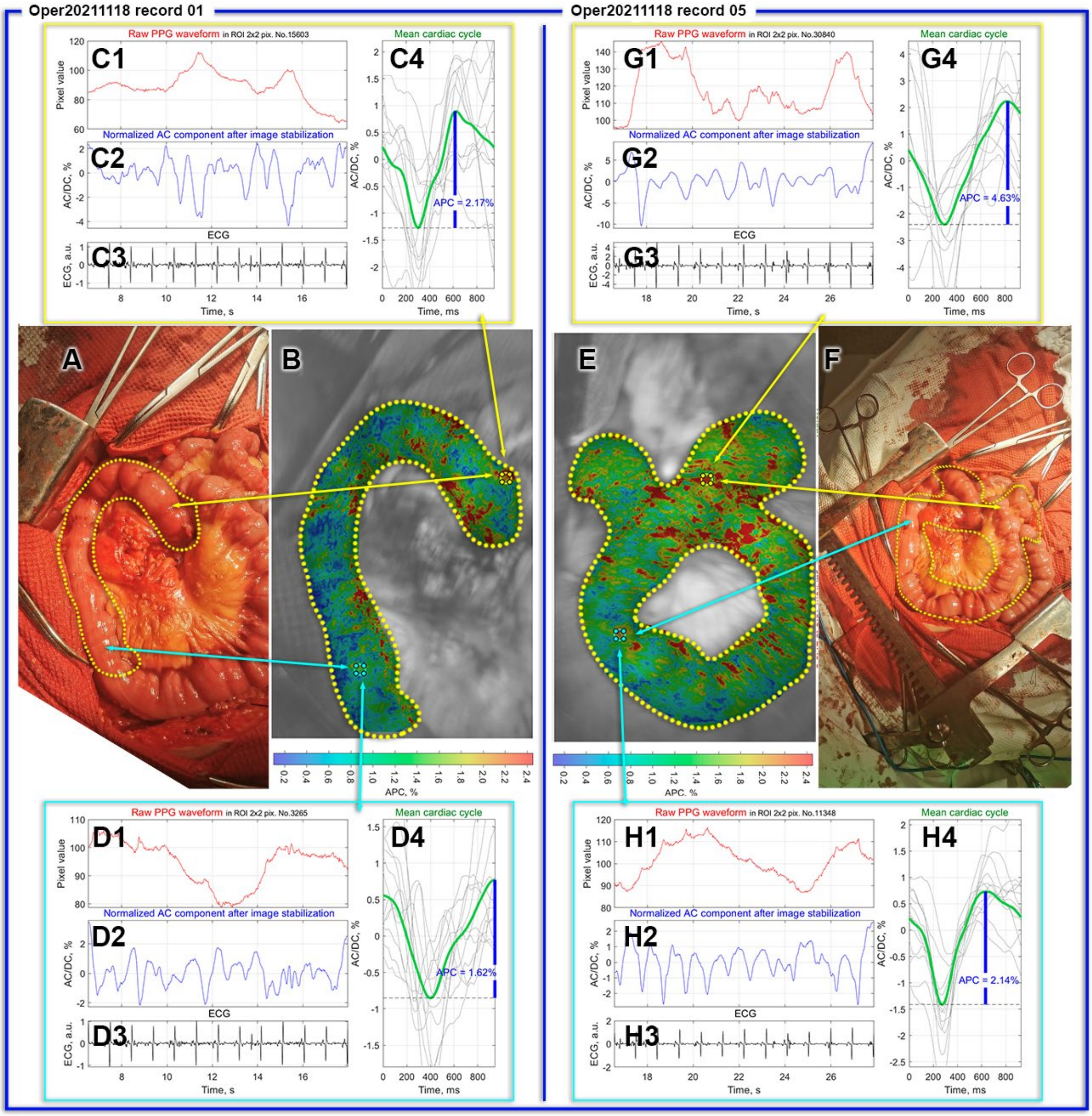

Figure S7. Blood perfusion visualization and examples of PPG waves. Diagnosis: Gastric cancer. Surgery: Total gastrectomy. Panels A and F are photographs of the patient's bowels before and after anastomosis, respectively. Yellow dashed lines in these panels indicate the areas within which mapping of the perfusion was assessed. Spatial distribution of the index perfusion APC before and after anastomosis are shown in Panels B and E, respectively.

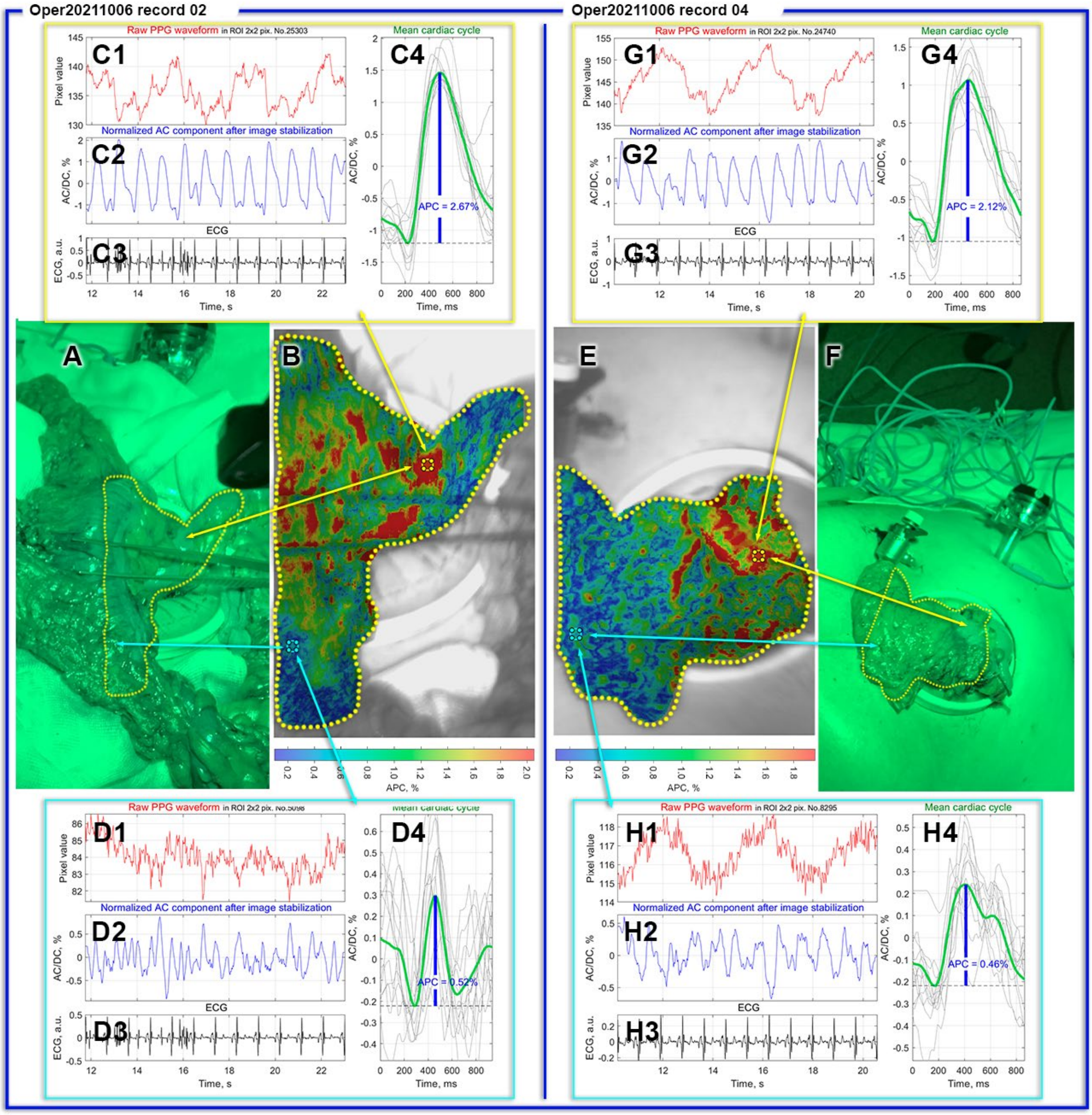

Figure S8. Blood perfusion visualization and examples of PPG waves. Diagnosis: Cancer of ascending colon. Surgery: Laparoscopically assisted right-sided hemicolectomy. Panels A and F are photographs of the patient's bowels before and after anastomosis, respectively. Yellow dashed lines in these panels indicate the areas within which mapping of the perfusion was assessed. Spatial distribution of the index perfusion APC before and after anastomosis are shown in Panels B and E, respectively.

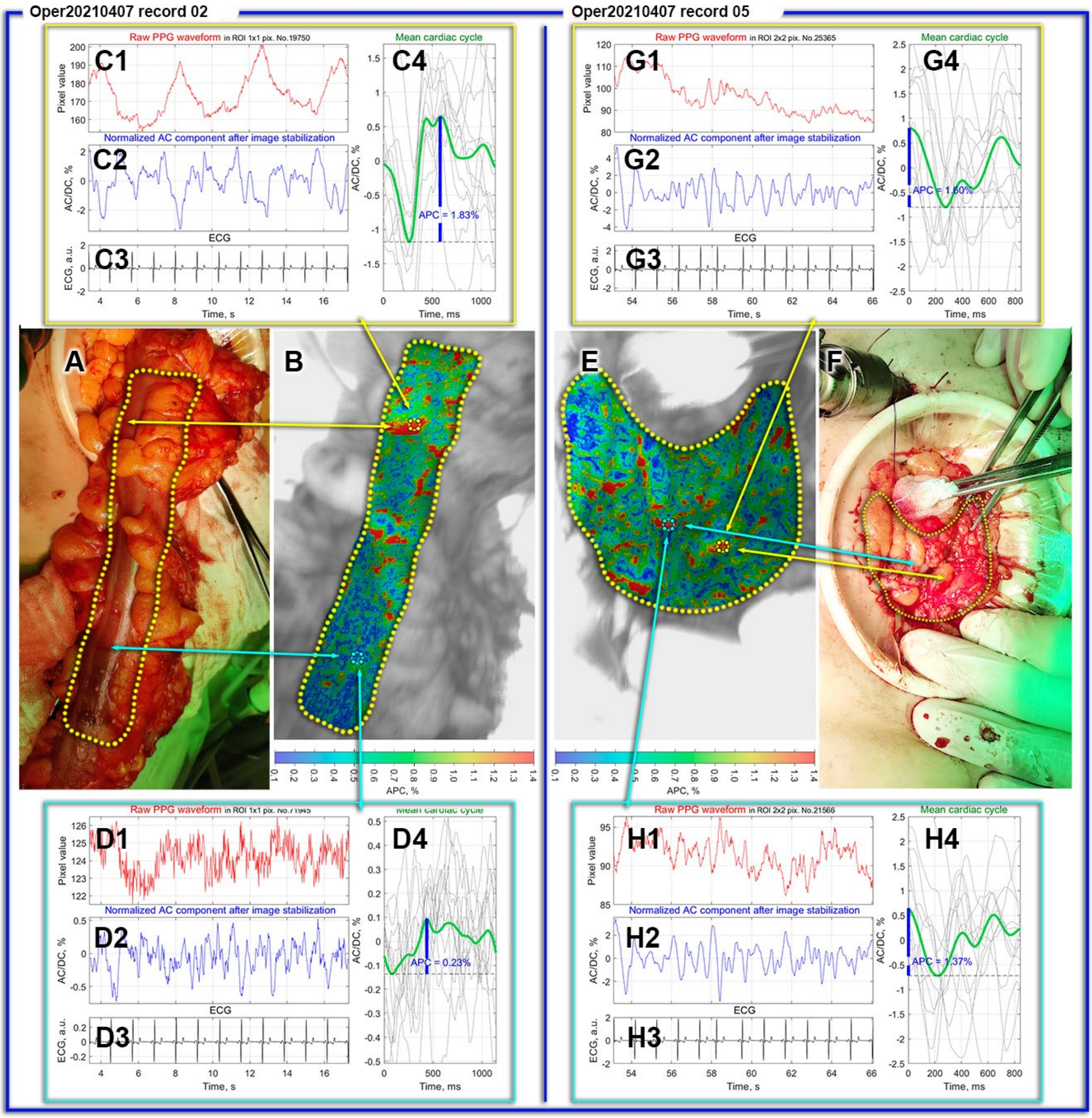

Figure S9. Blood perfusion visualization and examples of PPG waves. Diagnosis: Sigmoid colon cancer. Surgery: Laparoscopically assisted left colon resection. Panels A and F are photographs of the patient's bowels before and after anastomosis, respectively. Yellow dashed lines in these panels indicate the areas within which mapping of the perfusion was assessed. Spatial distribution of the index perfusion APC before and after anastomosis are shown in Panels B and E, respectively.

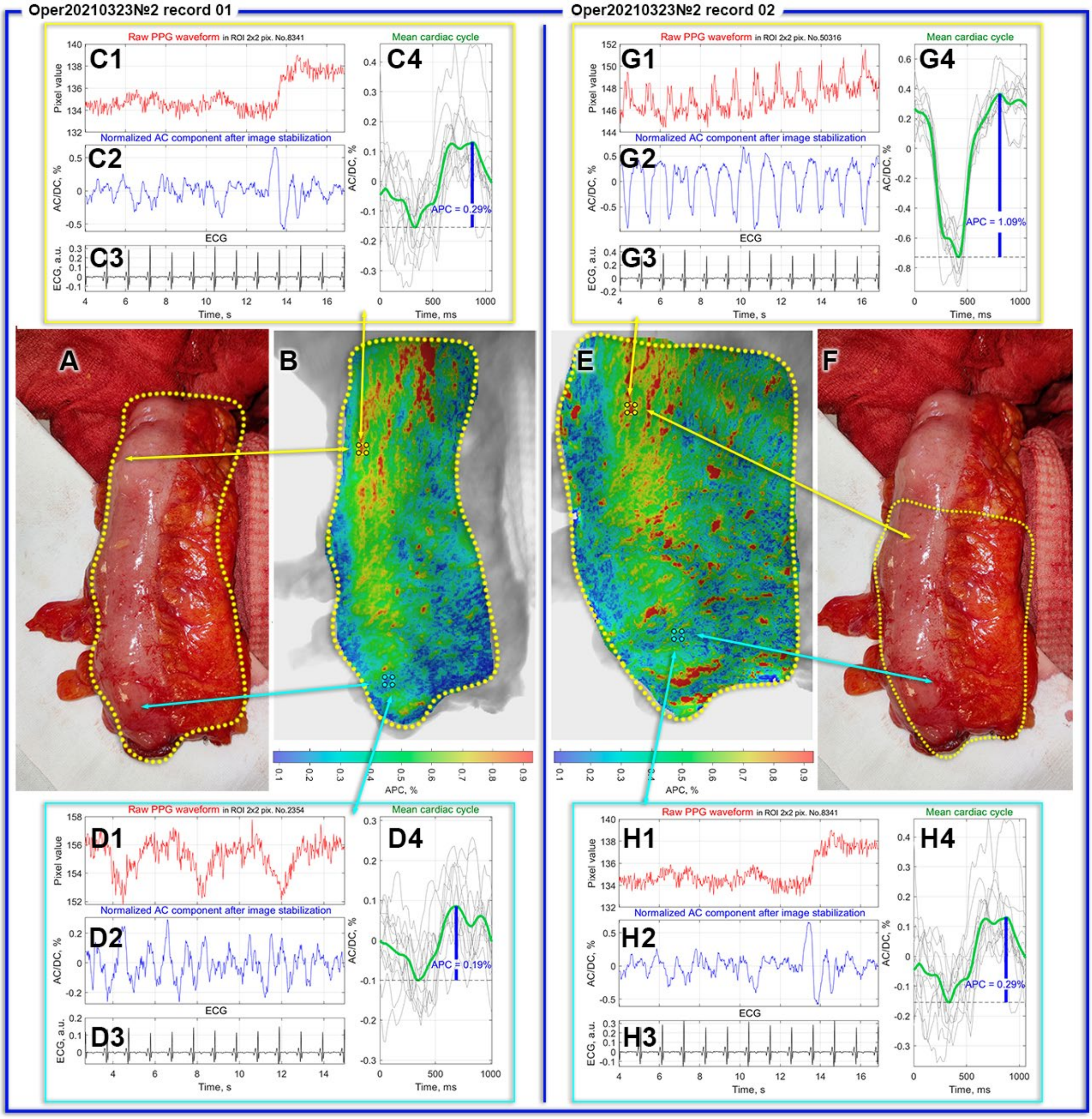

Figure S10. Blood perfusion visualization and examples of PPG waves. Diagnosis: Sigmoid colon cancer. Surgery: Laparoscopically assisted sigmoid colon resection. Panels A and F are photographs of the patient's colon. Yellow dashed lines in these panels indicate the areas within which mapping of the perfusion was assessed. Panel B shows spatial distribution of the index perfusion APC after organs mobilization but before anastomosis, while the panel E shows APC map of the same colon 15 minutes later.

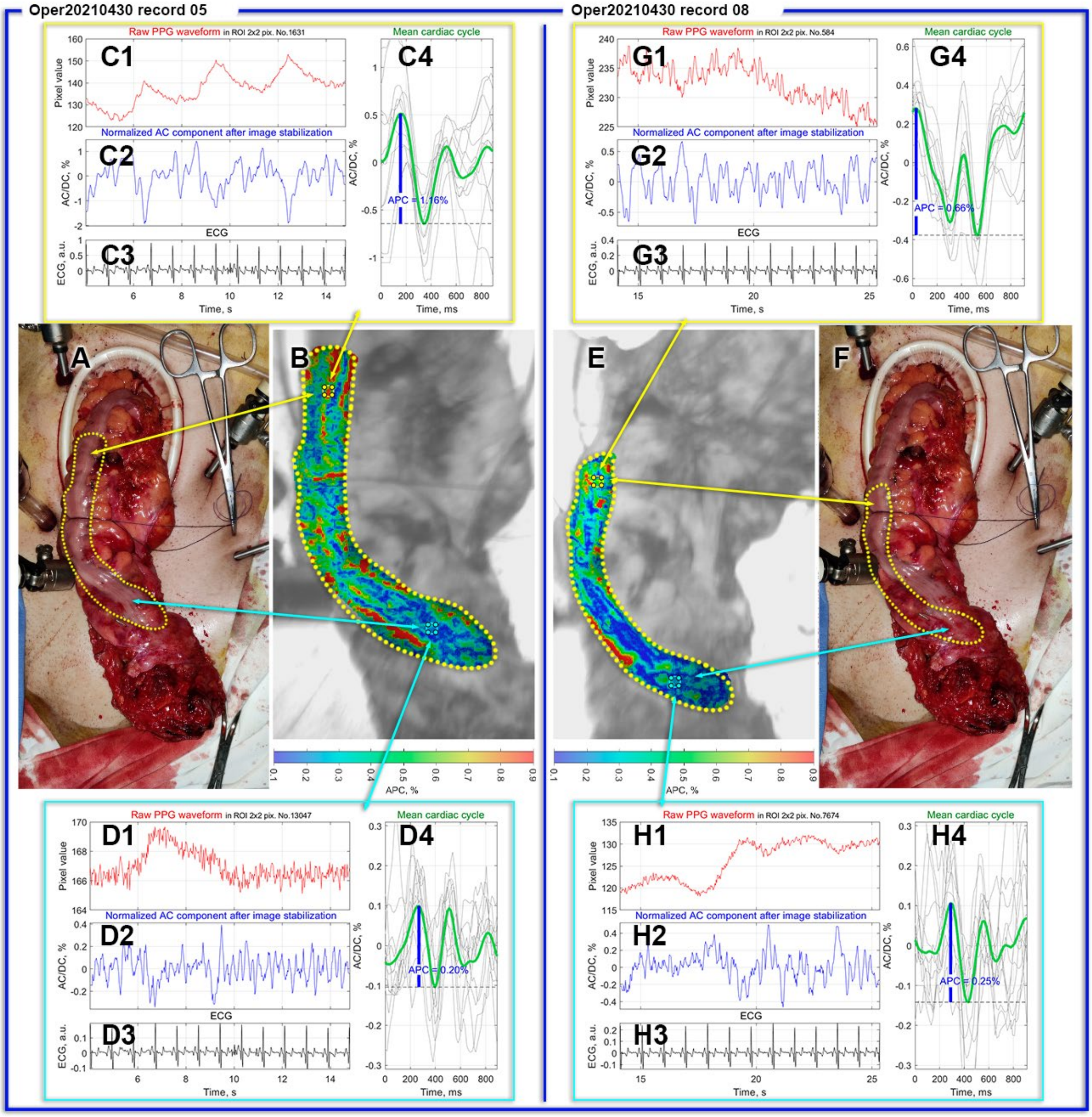

Figure S11. Blood perfusion visualization and examples of PPG waves. Diagnosis: Upper rectal cancer. Surgery: Laparoscopically assisted anterior rectal resection. Panels A and F are photographs of the patient's rectum. Yellow dashed lines in these panels indicate the areas within which mapping of the perfusion was assessed. Panel B shows spatial distribution of the index perfusion APC after rectum mobilization but before anastomosis, whereas the panel F shows distribution of the index perfusion over the same rectum but for limited blood supply due to the clamping one of the arteries. Mean perfusion index is 0.43 ± 0.30 % before clamping and 0.31 ± 0.20 % after it (see Table 1). Note the 30% decrease (p < 0.001) in perfusion index averaged over the entire APC mapping area.

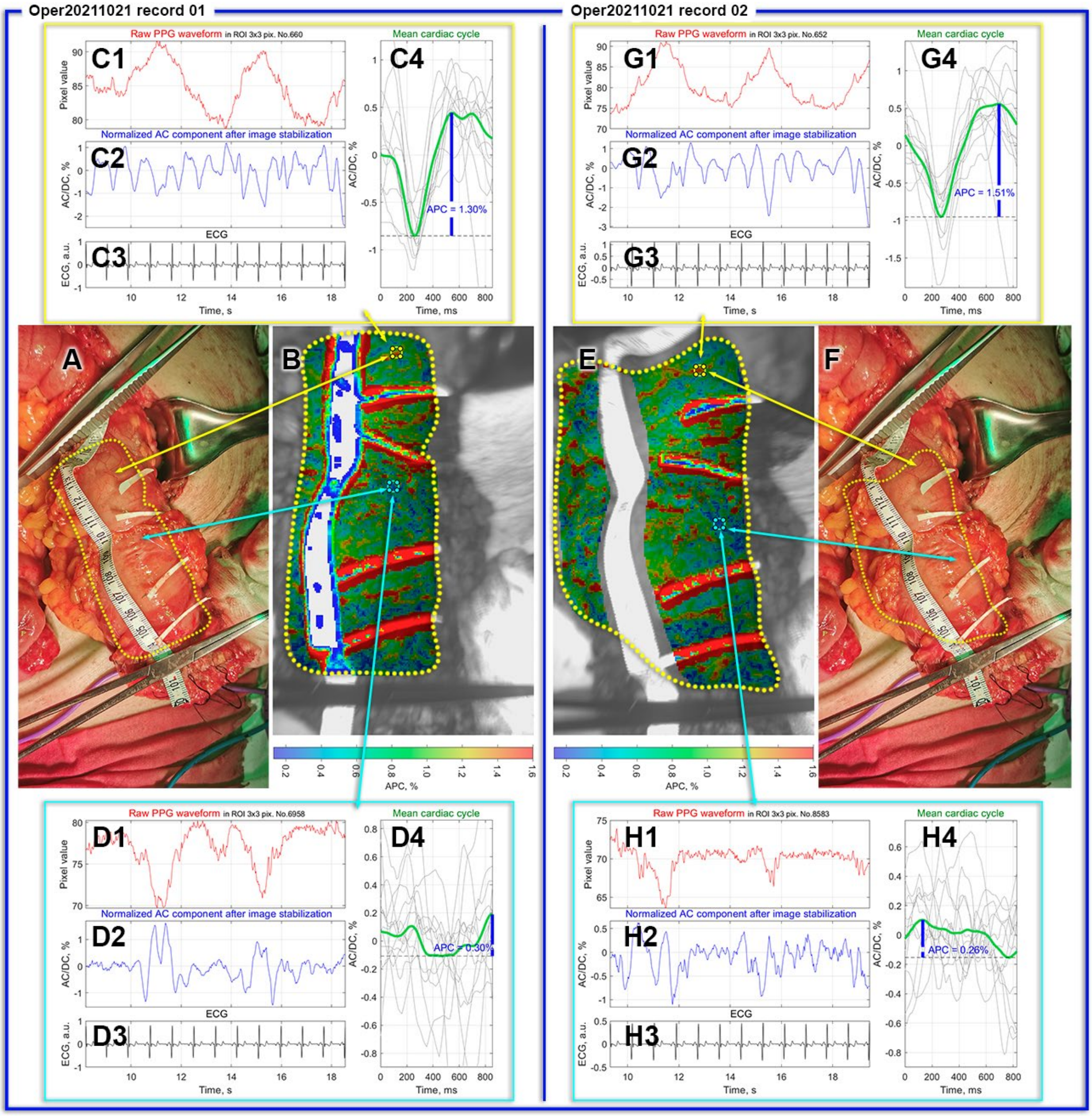

Figure S12. Blood perfusion visualization and examples of PPG waves. Diagnosis: Rectosigmoid cancer. Surgery: Descendorectostomy with ileostomy and hepatic resection. Panels A and F are photographs of the patient's colon. Yellow dashed lines in these panels indicate the areas within which mapping of the perfusion was assessed. Panel B shows spatial distribution of the index perfusion APC after organs mobilization but before anastomosis, while the panel E shows APC map of the same colon 2 minutes later.
